# All professions can benefit — a mixed-methods study on simulation-based teamwork training for operating room teams

**DOI:** 10.1186/s41077-023-00257-0

**Published:** 2023-07-17

**Authors:** Cecilia Escher, Hans Rystedt, Johan Creutzfeldt, Lisbet Meurling, Leif Hedman, Li Felländer-Tsai, Ann Kjellin

**Affiliations:** 1 Center for Advanced Medical Simulation and Training (CAMST), Karolinska University Hospital, Stockholm, Sweden; 2 Department of Clinical Science Intervention and Technology (CLINTEC), Karolinska Institutet, Stockholm, Sweden; 3 Simulator Centre West, Region Västra Götaland, Gothenburg, Sweden; 4 Department of Psychology, Umeå University, Umeå, Sweden

## Abstract

**Background:**

Operating rooms have become more technically complex due to new advanced procedures, which has increased demands on teamwork in the operating room. In response, team training has been proposed to improve team performance, workplace culture, and patient safety. We developed and delivered a simulation-based team training course for entire professional surgical teams. This type of intervention has been proposed by researchers but has not been widely published. The aims of this intervention study were to examine participants’ reactions to the course in terms of their motivation for the training and their self-efficacy in relation to their performance, as well as their views on transferring the lessons learned in the course to their workplace.

**Methods:**

In a prospective mixed-methods intervention study, operating room professionals participated in a full-day simulation-based teamwork training course. Learning objectives were nontechnical skills, specifically communication and collaboration across the team.

Seventy-one staff members representing 5 operating room professions were included, and the average work experience of participants was 6 years.

Quantitative data on self-efficacy and situational motivation were collected by questionnaires before and after training. Qualitative data were collected through 5 focus group interviews that took place in direct relation to the courses and included a total of 31 participants. Transcripts were coded and analyzed using thematic analysis.

**Results:**

All occupations showed a similar pattern in terms of increases in self-efficacy and intrinsic motivation after the training. Analysis of the qualitative data showed that training in one’s profession and in authentic multiprofessional teams was important factors for motivation. Participating staff described an awareness of undesirable communication barriers in surgical teams that can lead to risks for patients. Systematic training was definitely perceived as a means to reduce barriers and improve communication and collaboration.

**Conclusion:**

Simulation-based training was equally well received by all professional groups. Our results confirm the feasibility of this type of training for professional teams and promising opportunities for improving teamwork skills. The qualitative data reveal both opportunities and limitations for transferring the learning experiences to the workplace.

**Supplementary Information:**

The online version contains supplementary material available at 10.1186/s41077-023-00257-0.

## Background

Recognized problems related to patient harm during and after surgery have brought patient safety into focus in surgical and operating room (OR) departments [1, 2]. Surgical patients are vulnerable to a range of problems including unsafe anesthesia, infections, and technical mishaps. At the same time, margins are narrowing as patients with serious comorbidities present more frequently and the number of technically challenging procedures increases. Larger teams, often organizationally divided into an anesthesia subteam and a surgical subteam, make teamwork and coordination more complex. At the same time, the margins are narrowed as patients with severe comorbidities are presented more frequently and technically advanced procedures are increasing. The demand for good communication skills is large and increases further when staff in ORs for transplantation, robotic, and hybrid surgery are separated wide apart in huge theaters [3, 4]. Studies have shown that the quality of communication and the patient safety climate are perceived differently among staff working in ORs [5, 6]. In a study examining barriers to interprofessional teamwork in ORs, personality, hierarchies, gender, and lack of knowledge about teamwork were cited as important factors [7]. Shi et al. reached a similar conclusion but added uncordiality as a prominent barrier to communication in the OR [8]. Quality of communication was found to correlate positively with favorable surgical outcomes [9], whereas inadequate nontechnical skills were found to correlate with poorer outcomes [10].

Simulation-based teamwork training (SBTT) has been shown to be effective in improving teamwork [11, 12] and patient safety [13, 14] in a wide range of settings. Researchers have highlighted interprofessional simulation as an important educational intervention for graduate, postgraduate, and continuous professional development [15, 16].

In perioperative care, the WHO surgical safety checklist has been successful in reducing errors [1, 13, 17–19] and improving communication and collaboration [20] and is now part of standard care in ORs around the world. Other patient safety initiatives have followed, covering the entire care pathway for surgical patients [21, 22].

Brief in situ simulations of OR teams have shown improvement in nontechnical skills [23], as have classroom-based approaches [24, 25]. Nevertheless, there are few studies on the effects of SBTT on authentic OR teams; one review included only 10 studies [26].

We designed and investigated a SBTT course for full professional OR teams based on a pilot project [27]. To gain insight into professionals’ individual responses to the course, we estimated two known motivational factors before and after the training: participants’ self-efficacy and their situational motivation.

Self-efficacy or self-confidence is the optimistic belief in one’s own competence or chances of successfully completing a task [28]. If a person does not believe in his or her ability to achieve a goal, he or she will have less motivation to exert effort. We were interested in self-efficacy because it correlates with performance in a number of situations [29], and we have previously found that SBTT leads to higher self-efficacy scores [12].

Situational (or state) motivation provides a valuable assessment of an individual’s current self-regulatory processes. Professional participants are often reluctant to practice, so we were interested in monitoring the motivation of our teams and individuals. In a pilot project, participants showed increased intrinsic motivation following a similar intervention [27].

The aims of this study were twofold: First, to examine the responses of the professional groups involved in the training, particularly issues related to the development of self-efficacy and situational motivation. In this particular setting, we were also interested in commonalities and differences regarding occupation-specific responses to the course. Second, we wanted to explore participants’ perceptions of the design features important to the training and the opportunities for and barriers to transferring the lessons learned in SBTT to teamwork in the operating room.

## Methods

The design was a mixed-methods intervention study that included validated questionnaires to collect quantitative data on participants’ individual responses to SBTT and focus group interviews to collect qualitative data on perceptions of the training and opportunities for transfer of learning. Ethical approval was obtained from the regional ethics committees in Stockholm 358–02, 2017/2456–31/5, and Linköping 2012/439–31. Participation was voluntary, and informed consent was obtained from all participants.

### The training

A SBTT course for professional OR teams was developed and was well received in a pilot study [27]. The course lasted a full day and included the following: introduction, familiarization with the simulators and environment, scenario briefing, and 3–4 scenarios, each followed by a video-enhanced debriefing. The scenarios were parts of operations rather than complete procedures to avoid extended periods without tasks for the professionals involved. Episodes were selected when team interaction was intense. To ensure relevance to all, it was necessary that all professional groups were represented in the team of trainers. Each team consisted of 5–7 participants: resident surgeon, resident anesthetist, operating room nurse, nurse anesthetist, and associate nurse. The simulated events were emergencies during laparoscopic procedures, including failed intubation, pneumothorax, cardiac arrest, allergic reaction, hypotension, and uncertainty regarding patient identity. The introduction adressed nontechnical skills applicable in this setting and the WHO surgical safety checklist. Training objectives were derived from the concept of crew resource management (CRM) [30], which was refined in the ATEAMS program [31], and each scenario was followed by a video-enhanced debriefing focused on the training objectives. The simulators used were SimMan 3G (Laerdal, Norway) and LAP Mentor or LAP Mentor Express (Simbionix, Israel). There was extensive experience with SBTT in the group of trainers, in which all professional groups were represented.

### Participants

Eleven courses were held, involving 21 physicians (11 resident surgeons and 10 resident anesthetists) and 37 nurses (20 nurse anesthetists and 17 operating room nurses and 13 associate nurses). Participants were recruited from 2 large Swedish OR departments performing general and laparoscopic surgery. Staff members were trained in their respective teams.

### Quantitative data

#### Background data

Data on occupation, sex, age, and work experience were collected at the beginning of the course. The data are presented in Table 1.

**Table 1:** Background data

Sex	Male 17 (24%), female 55 (76%)
Age in years mean (range)	39 (22–55)
Experience on the job in years mean (range)	6.1 (0.4–29)

#### Self-efficacy (SE)

After the course introduction, but before the first scenario and at the end of the course, data on self-efficacy were collected. The SE questionnaire [32] was modified, translated, and validated for the Swedish setting by one of the authors (L. H.) and contained 5 items on participants’ self-efficacy beliefs about the training. Each item was rated by the participants on a 7-point Likert scale, and a mean SE score was calculated.

#### Situational motivation

Situational motivation data were collected after the introduction but before the first scenario and at the end of the course. Situational motivation (or state motivation) provides a valuable assessment of a person’s current self-regulatory processes. The Situational Motivation Scale (SIMS) measures motivation at a particular point in time. It captures four types of human motivation as described in self-determination theory [33–35]. Intrinsic motivation captures involvement in a task of one’s own volition and interest, for its own sake. Internal regulation refers to tasks done out of the belief that they will lead to some kind of personal reward, so the motivation comes “from within.” The aforementioned types are also classified as autonomous. External regulation stimulates us to do tasks because of an external influence. Amotivation is when we do not understand the aim and purpose of performing a task. The instrument has been translated and validated in the Swedish context by one of the authors (LH) and contains 4 questions about each type of motivation, rated on a 7-point Likert scale [33].

### Qualitative data

#### Focus group interviews

Semi-structured focus group interviews were conducted on five occasions after the course, with an entire team participating each time. The interview guide was developed by CE, JC, and LH and included open-ended questions about participants’ perceptions of the training. Interview data were collected and analyzed to answer the second research question, participants’ perceptions of the design features important to training and the opportunities, and barriers to transferring lessons learned in SBTT to teamwork in the operating room. The interviewer (CE) did not know any of the participants before the course began but was one of the facilitators during the course. The first 5 teams to participate in the study were interviewed. Thirty-one of the 32 participants agreed to participate in the interviews, which were conducted after the conclusion of the course. The interviews, which were videotaped, lasted 20–40 min and included 5–7 participants each. Field notes were taken to allow for identification on the video files.

After a preliminary analysis of the 5 interviews, each of which included representatives from the 5 professions, the data were considered saturated [36].

#### Analysis of the focus groups

Assistants transcribed the recordings verbatim, and CE and HR reviewed the transcriptions against the recordings. Thematic analysis was conducted following the method described by Braun and Clarke [37]. Two researchers (HR and CE) coded separately and found preliminary subthemes and themes in a process guided by the research questions. In the next step, preliminary themes were negotiated in an iterative process until agreement on themes and subthemes was reached. Representative quotes were selected and translated into English.

#### Statistical methods and data management

To allow comparisons between the relatively small professional groups, quantitative data were analyzed in two ways. First, surgeons and anesthesiologists, operating room nurses, and nurse anesthetists were combined into one physician and one nurse group, with associate nurses forming a separate group. Second, OR nurses, surgeons, and associate nurses were combined into a surgery subteam, and anesthesiologists, nurse anesthetists, and associate nurse anesthetists were combined into an anesthesia subteam. These groups formed the units for statistical analysis. One participant inadvertently participated twice in the study; data from his second encounter were excluded. Missing values were 1–6% of the total number of responses.

Multiple comparisons of continuous data were performed using analysis of variance, ANOVA. In the case of a statistically significant result in the ANOVA, statistical comparisons were performed using the post hoc test proposed by Fisher to control for multiplicity. Statistical comparisons to test differences between two independent groups were performed using Student’s *t*-test for uncorrelated means. Differences between two dependent measurements were assessed with Student’s *t*-test for correlated means. The Pearson correlation coefficient was used to test independence between variables. In addition, descriptive statistics were used to characterize the data. All analyses were performed using SAS statistical software, with a significance level of 5%. In case of a statistically significant result, the probability value (*p*-value) was given.

## Results

### Quantitative analysis

We examined data from 69 participants regarding self-rated self-efficacy and situational motivation before starting the first scenario and immediately after participation in a full-day SBTT course. Both self-efficacy and situational motivation scores improved significantly after the training. To analyze the responses of each professional group, data were also grouped by hospital, subteam, experience, and profession.

#### Self-efficacy

SE scores increased after training, SE before: mean 5.0 (*SD* 1.0) and SE after: mean 5.9 (*SD* 0.7) *p* < 0.0001. No significant differences were found in self-efficacy scores after training with respect to occupations, the two participating hospitals, staff experience, or subteam membership (Table 2).

**Table 2:** SE after training per group n, mean (SD)

Hospital	1 *n* = 31 5.9 (0.7)	2 *n* = 38 5.9 (0.6)		Ns
Sub-team	Anesthesia *n* = 33 5.7 (0.7)	Surgical *n* = 36 6.0 (0.6)		Ns
Experience	< 5 years *n* = 33 5.8 (0.7)	> 5 years *n* = 34 6.0 (0.6)		Ns
Profession	Doctor *n* = 21 6.0 (0.7)	Registered nurse *n* = 35 5.8 (0.7)	Associate nurse *n* = 13 5.7 (0.5)	Ns
All	*n* = 69 5.9 (0.7)			

#### Situational motivation and engagement

Quantitative analysis revealed that situational motivation in the form of intrinsic motivation (IM) and identified regulation (IR) increased significantly after training: SIMS IM before: mean 5.2 (0.9) and SIMS IM after: mean 5.8 (0.9), *p* < 0.0001 and SIMS IR before: mean 5.6 (0.8) and after: mean 5.8 (0.8), *p* = 0.013. Amotivation (AM) showed a negative trend, with a change in AM of − 0.3 (0.7) *p* = 0.003. Intrinsic motivation scores after training did not differ between groups (Table 3). There was a significant difference in the increase in intrinsic motivation between the surgical and anesthesiology subteams, with the OR subteams showing a greater change (*p* = 0.028), but the post-training scores did not differ.

**Table 3:** Intrinsic motivation after training per group n, mean

Hospital	1 *n* = 30 5.8 (0.8)	2 *n* = 40 5.8 (1.0)		Ns
Team	Anesthesia *n* = 34 5.6 (1.0)	Surgical *n* = 36 6.0 (0.8)		Ns
Experience	< 5 years *n* = 33 5.7 (1.0)	> 5 years *n* = 35 5.9 (0.9)		Ns
Profession	Doctor *n* = 21 5.6 (0.8)	Registered nurse *n* = 36 5.8 (1.0)	Associated nurse *n* = 13 6.1 (0.8)	Ns
All	*n* = 70 5.8 (0.9)			

### Qualitative analysis

Five teams took part in focus group interviews after training. Thematic analysis of the transcripts resulted in three themes: belief in one’s own abilities, engagement, and transferability. Each theme includes 3–4 subthemes illustrating each theme more specifically (Table 4).

**Table 4:** Overview of themes and subthemes from the qualitative analysis

Themes	Subthemes
Belief in one’s own abilities	Setting and feedback Professional role Apprehension and reinforcement of self-efficacy
Engagement	To immerse in the situation Perception of knowledge gap Realism
Transferability	Understanding each other’s professional roles Insights into the principles of teamwork Opportunities and barriers to transfer The barrier in the OR

#### Belief in one’s own abilities

This first theme includes three subthemes that identify factors that contribute to participants’ self-belief in this setting and factors that contribute to low self-efficacy.

##### Setting and feedback

Many participants reported feeling anxious and fearful before the course, especially staff who had never participated in SBTT before. They feared being observed, not performing well, and experiencing the unfamiliar environment. Participants noted a strengthened confidence in their own abilities which developed over the course of the day. A number of reasons were cited such as feedback and learning new tools to improve teamwork.* “The difference is also that I get feedback after the scenario. If I did well, it’s a confirmation that my day-to-day performance is OK, and now I have tools that can lead to further improvement”* (male anesthesiologist, interview 3).

##### Professional role

Participants valued training in their own professional role with staff from their own OR as a reason for increased belief in their own abilities, compared to courses where this was not the case: *“Here you (the trainers) were very clear that we were expected to act in our own professional role, … before that, I was supposed to act as an anesthesiologist in an emergency exercise, and then… I felt very incompetent”* (female surgeon interview 4).

##### Apprehension and reinforcement of self-efficacy

Staff expressed that the training was an affirmation of their own professional competence: *“I know how to act in my job as an OR nurse …I grow when I overcome the challenges in the scenarios”* (female OR nurse, interview 1).

#### Engagement

This second theme includes three subthemes that identify factors that contribute to participants’ motivation to participate and engage in the scenarios. The subthemes range from recognizing the importance of reflection after the scenarios to how fidelity of the scenarios can contribute to or, in some cases, be a barrier to engagement.

##### To immerse in the situation

Participants expressed that they became increasingly engaged in the scenarios as they became more familiar with the situation. Some indicated that they sometimes forgot that the situation was not real. *“When the patient’s condition deteriorated, I was stressed, it kept me busy because I was immersed in the situation”* (female surgeon, interview 4).

##### Perception of knowledge gaps

The importance of effective teamwork and an increased awareness of gaps in teamwork knowledge and skills were expressed. Participants related the SBTT to their experiences working at OR: *“We work in teams a lot, but we never think about how…. of course, it’s an important part of the work environment and the quality of work”* (male surgeon, interview 1). Compared to work, the opportunity to reflect after SBTT scenarios was valued to identify knowledge gaps. *“…getting feedback with a video is very helpful, seeing my own behavior and our behavior as a team and getting feedback is really important”* (female OR nurse, interview 1).

##### Realism

The design and realism of the SBTT were cited as helpful in increasing engagement. Participants repeatedly expressed that working in their own professional roles and acting as a whole OR team enhanced their engagement, *“…if the anesthesia staff had not participated, it would not have felt as real”* (female OR nurse, interview 1). Participants also expressed that the authenticity of the simulators and other equipment was important to their motivation, but sometimes when information was missing, they felt the setting was a limitation. *“I sometimes felt removed from realism when I did not know where to find the information I needed”* (male surgeon, interview 1).

#### Transferability

This third theme includes four subthemes that demonstrate how participants’ learning from the course can be used in the workplace but also how the existing organization of teamwork in their normal OR might be a barrier to using the learning from the course.

##### Understanding each other’s professional roles

The opportunity to gain new insights and discuss tasks, professional roles, and interdependence was highlighted repeatedly in the interviews:

*“... we are totally dependent on each other’s competence, otherwise we would not be able to do the work we are supposed to do, the understanding of the other professions’ areas increases immensely when we train like this. What is important for one profession is important for the others. It’s the foundation for patient safety and also for our confidence, it’s really important to do this kind of training, I think”* (female OR nurse, interview 1).

Others expressed new insights and a willingness to assist and ask for support in difficult situations in the future:

*“Sometimes you think, this is my thing and I’ll figure it out and it’s hard when I have a problem. And then you forget a little bit about the competencies of others when you’re talking, what does anesthesia think? I think that is the most important aspect of training, teamwork with other clinicians”* (female surgeon, interview 4).

##### Insights into the principles of teamwork

The tools of teamwork were discussed, and taking a time-out was seen as an important aid to assess the situation, improve situational awareness, and to break down professional barriers. *“It seems that time-out is a very good way to reduce the barrier between anesthesia and surgery”* (female nurse anesthetist, interview 1). *“I think I will try to push more for a time-out, not just the younger surgeons who are already doing it. …it's also important when we change staff in the afternoon”* (female associate nurse, interview 2). Furthermore, situational awareness for the entire team, reducing the barrier between the sterile field and anesthesia, was pointed out as important lessons learned.

*“It is routine to have a time-out, and I feel like I know how things are on the other side…but now I realize that I should pay more attention, it´s easy to focus and just occasionally ask, how the patient is doing”* (female surgeon, interview 4)

##### Opportunities and barriers to transfer

Organizational issues were cited as important barriers to behavioral change in the workplace. Participants were concerned that only some of the entire staff were trained, and the rest did not have access to the same tools. Participants also asked for support from management to improve communication in the OR. *“…It would be a big help if we were all trained like that…we could implement regular time-outs as a quality indicator” (male nurse anesthetist, interview 1). *Staff also expressed anticipated difficulties and frustrations in changing existing behaviors in their usual work:* “..we have been given tools to use both as a team and as individuals, but then other members may not listen and understand, so that can be quite frustrating “ *(female OR nurse, interview 1).

##### The barrier in the OR

Participants repeatedly spoke about the prevailing barrier in OR, referring both to the shielding of the sterile field and the divide between the anesthesia and surgical sub-teams. *“The shielding between anesthesia and surgery gets a little lower (after training), it would be great if it was not watertight” (male anesthesiologist, interview 2). Staff members expressed that they valued interprofessional dialog because they had few joint meetings in their workplace and rarely met with members of other professions outside of OR. The WHO surgical safety checklist was cited as an important tool for teamwork because it promotes communication throughout the team. Staff also cited the barrier in the OR as a potential threat to patient safety: “… more dialog through the screen is important to improve communication and increase patient safety”* (female nurse anesthetist, interview 3).

## Discussion

Our quantitative analysis showed that experienced employees from 5 professions working together in a OR increased their self-confidence and intrinsic motivation in a similar pattern across professions after a SBTT intervention. Qualitative data indicated that staff saw both opportunities and challenges in transferring their learning experiences from SBTT to operating rooms.

When given the opportunity to discuss teamwork in interprofessional teams, perceptions of communication barriers in the OR were revealed, and SBTT was perceived as a means to improve teamwork, reduce barriers, and strengthen patient safety.

Previous studies, like this one, have shown improvement in self-efficacy scores after teamwork training [12, 38]. This study adds a qualitative analysis highlighting how the features and effects of the training helped to increase self-confidence. Training in one’s own professions and in a complete authentic team, as well as receiving feedback, was cited as important factor for positive changes in self-belief.

Employees in our cohort were highly engaged in the training, and scores on autonomous aspects of situational motivation increased significantly after the training. To our knowledge, situational motivation has not been extensively studied in research in this area [27]. In a previous study, the flow experience of team leaders, which is known to be a strong motivator, was significantly higher than that of followers [39], so we would have expected that physicians who serve more frequently as team leaders would be more motivated to train. One reason for our results showing that the occupational groups had similar scores regarding autonomous motivation after training might be that the groups were too small to detect a true difference. On the other hand, the analysis of our qualitative data speaks to the applicability of the course regardless of occupation. Our interpretation, therefore, is that a well-designed course can appeal to all occupational groups.

The rationale for SBTT is the ability to improve both technical and nontechnical performance in real-world clinical settings; therefore, staff perceptions of the transferability of skills from SBTT to OR were of particular interest. Transferability was a major theme that emerged from the qualitative analysis and included both opportunities and limitations. The importance of using tools such as a time-out and more conscious and consistent use of the WHO surgical safety checklist were cited as impacts of training in the clinical setting. The opportunity to reflect on challenges of working in a team was highly valued. Perceived barriers to transfer in our study included colleague resistance to change and lack of organizational support. These barriers are consistent with previous research by Salas et al. who identified culture and context as two important aspects of maintaining and improving teamwork in all organizations [40]. There was a difference in seniority among our participants, with nurses generally being highly experienced and physicians being residents. This situation is not uncommon in our ORs, and interestingly, this limitation was not highlighted in the interviews. However, the difference in experience is a limitation in the interpretation of our results because consultants have a large influence in the ORs.

Interestingly, in the analysis of the qualitative data, the OR was described as being divided by the screen between anesthesia and the sterile surgical field. Our interpretation of this barrier was both the physical aspect of a barrier and a metaphor for professional communication barriers in the OR. The screen as a metaphor for professional barriers is consistent with the work of Makary et al. on differences in perceptions of the quality of collaboration depending on profession OR [5]. In Makary’s study, employees at higher hierarchical levels perceived the quality of teamwork more positively than employees at lower hierarchical levels [5], and others have found similar patterns [6, 7]. Staff in our study requested opportunities for discussion and collaborative practice and acknowledged interdependence in the multiprofessional team for safe and effective performance. Our findings are consistent with the ongoing debate challenging the prevailing organization and training of healthcare professionals in separate silos [15] and emphasizing the importance of competent teamwork for safe care.

Many questions remain about the best practice of SBTT, such as how often and how long a session is most effective. This study confirms the feasibility of SBTT for experienced OR teams, a type of intervention that has been proposed but, to our knowledge, not often implemented or published [26].

The mixed-methods design allowed conclusions to be drawn beyond self-assessed values. In summary, all professional groups responded very well to the training. Practicing teamwork tools, gaining new insights into the roles of other professional groups, and discussing teamwork in a multidisciplinary team were some of the benefits.

Awareness of inadequate teamwork as a cause of patient harm, differing perceptions of patient safety across professional groups, combined with increasing technical challenges and larger teams, is putting pressure on OR departments and staff. A SBTT course, such as the one described in this study, offers a promising opportunity to improve teamwork and enhance patient safety.

### Limitations

The study was conducted at a single Swedish simulation center but included participants from two hospitals of similar size. The small number of participants was a limitation to the quantitative analysis, potentially masking true differences between the groups. The ceiling effect may also have played a role, as scores were already relatively high before training. Participants were not homogeneous in terms of work experience and gender; women dominated, but this reflects the situation in many OR departments. In addition, we included experienced residents but not specialists for organizational reasons. Residents are the specialists of the future, but the longer average work experience of nurses compared with physicians in the study may have influenced the results. The focus group interviews were limited to participants from one of the hospitals, and CE conducted the interviews and served as one of the facilitators of the courses. However, HR participated equally in the qualitative analysis as a learning sciences researcher independent of the center, limiting the risk of bias.

## Conclusion

Team training for experienced complete OR teams was feasible, and self-confidence and intrinsic motivation increased regardless of profession. Training in authentic complete teams and receiving feedback were important factors. Professional communication barriers at ORs were identified as a risk to patient safety, and our findings indicate that SBTT has the potential to improve nontechnical skills, promote interprofessional dialog, reduce professional barriers, and improve patient safety. Experienced facilitators from each of the five professions and careful development of scenarios with procedures requiring team communication were considered key factors in this training. Challenges in transferring what was learned to ORs included organizational barriers.

## Supplementary Information

**Additional file 1.** Dataset.

**Additional file 2.** Scenario 1: Wrong patient ID and hypotension during laparoscopic cholecystectomy. Scenario 2: Pneumothorax during laparoscopic cholecystectomy.

## Data Availability

The quantitative dataset analyzed in this study is available as a Supplement. The qualitative dataset is not publicly available but can be accessed from the corresponding author in the original Swedish version upon reasonable request.

## Abbreviations

SBTT: Simulation-based teamwork training
IM: Intrinsic motivation
IR: Identified regulation
AM: Amotivation
CRM: Crew resource management
OR: Operating room
WHO: World Health Organization
SIMS: Situational motivation scale
SE: Self-efficacy
ATEAM: All team member program
