# Supplementary material for: All professions can benefit — a mixed-methods study on simulation-based teamwork training for operating room teams
Source: Adv Simul (Lond). 2023 Jul 17;8:18. doi: 10.1186/s41077-023-00257-0 (PMC10351117; doi:10.1186/s41077-023-00257-0)
Supplement: Supplementary file 2 — Additional file 2. Scenario 1: Wrong patient ID and hypotension during laparoscopic cholecystectomy. Scenario 2: Pneumothorax during laparoscopic cholecystectomy. [file 41077_2023_257_MOESM2_ESM.docx]

**Scenario 1: Wrong patient ID and hypotension during laparoscopic cholecystectomy**

The patient is 40 years old and suffers from hypertension.

The trainees have time to read the patient's chart and preoperative assessment before handover.

The trainer team hands over to the trainees after induction of anesthesia and preparation of the sterile field. The patient is monitored, intubated, and the surgical field is prepared (the surgeon has a family emergency as an excuse to leave). Each trainer hands over to their colleague and is given time to explain what has been done and the status of the patient, medications, and equipment.

The instructors retire after the handover and are available to the team if they need clarification on the technical aspects of the simulation.

The patient wears an identification wrist band BUT ID does not match the records, anesthesia charts, or blood bank documents given to the anesthesiologist and nurse anesthetist.

Expected action:

1. During the time out before the procedure begins, the team needs to identify the mix-up, call the first surgeon and the anesthesiologist, and clarify which patient they have.

2. Begin the procedure.

When the bed is tilted up, the patient will have a sustained hypotensive episode.

Expected action:

1. Give medications to increase the blood pressure.

2. Tilt back the bed.

The scenario ends when the patient is stable.

**Scenario 2: Pneumothorax during laparoscopic cholecystectomy**

The patient has COPD.

The handover and preparations are the same as in the first case, but this time the surgery is almost complete at the time of handover.

The patient's breathing deteriorates and he develops a pneumothorax (due to emphysema, the first (i.e., trainer) anesthesiologist/nurse anesthetist performed some recruitment maneuvers before the handover due to the increased oxygen demand)

The pneumothorax develops into a tension pneumothorax with severe hypotension

Expected action:

1. Alert the team

2. Increase oxygen

3. Call for help

4. Prepare and perform decompression of the pneumothorax.

5. Prepare for insertion of a chest drain.

The scenario ends either just before or after the chest drain is inserted.
